# Performance assessment of three simplified Gielis equations in quantifying the geometries of lanceolate bamboo leaves

**DOI:** 10.3389/fpls.2025.1625685

**Published:** 2025-08-01

**Authors:** Qinchao Fu, Jing Li, Azuo Jimu, Ximeng Xiao, Lin Wang

**Affiliations:** ^1^ Key Laboratory of Sichuan Province for Bamboo Pests Control and Resource Development, Leshan Normal University, Leshan, China; ^2^ College of Life Sciences, Sichuan University, Chengdu, China

**Keywords:** close-to-linear behavior, goodness of fit, *Indocalamus*, leaf shape, relative curvature measures

## Abstract

Accurate quantification of bamboo leaf morphology is essential for understanding plant morphogenesis and development. However, most bamboo leaves exhibit long lanceolate shape characteristic, posing challenges in finding suitable mathematical models for accurate shape description. Previous studies indicated that the simplified versions of Gielis equation, a nonlinear polar coordinate system derived from the superellipse equation, have shown promise in describing bamboo leaf geometries. Nevertheless, selecting an optimal nonlinear equation that precisely fits empirical bamboo leaf data remains a formidable challenge in morphological studies. This persistent limitation underscores the critical need for developing systematic evaluation methods to assess the performance of such nonlinear models. In the present study, three distinct versions of simplified Gielis equation, i.e., four-parameter version (referred to as SGE-1), three-parameter version (referred to as SGE-2), and two-parameter version (referred to as SGE-3), were used to fit the two-dimensional contours of bamboo leaves with a long lanceolate shape across two species (*Indocalamus decorus* with 254 leaves, and *Indocalamus longiauritus* with 251 leaves). The root-mean-square error (RMSE) and Akaike information criterion (AIC) were employed to assess the goodness of fit and model structural complexity, and the nonlinear behavior for each model was assessed using relative curvature measures of nonlinearity. Across both datasets, SGE-1 showcased the lowest RMSE and AIC values but exhibited the poorest close-to-linear behavior based on relative curvature measures among the three models. Conversely, SGE-3 had the best close-to-linear behavior among the three models, but it exhibited the highest RMSE and AIC values. These findings provide a methodological framework for selecting nonlinear models in plant morphometrics, particularly for lanceolate-shaped leaves, while highlighting the critical balance between descriptive accuracy and statistical robustness in biological shape analysis.

## 1 Introduction

The leaf is the primary photosynthetic organ in most plants (Wright et al., 2004), and its morphology significantly influences plant growth and nutrient transport (Daas-Ghrib et al., 2010). As a result, an increasing number of mathematical models, such as the superellipse equation, have been developed to capture the geometric shape of leaves (Gielis, 2003). Bamboo, an essential component of ecosystems, is widely distributed in tropical and subtropical regions. Most bamboo leaves exhibit a long lanceolate shape, posing challenges in finding suitable mathematical models for accurate shape quantification. Fortunately, a polar coordinate equation was proposed by Gielis (2003), referred to as the Gielis equation hereinafter, which can effectively describe the two-dimensional contours of bamboo leaves, offering a promising approach for geometric modeling (Lin et al., 2016; Shi et al., 2018; Yao et al., 2022).

Gielis (2003) extended the superellipse equation to model a wide range of geometric shapes found in plants, which is usually reparameterized in the following form (Shi et al., 2020; Tian et al., 2020):


(1) $$r(\theta )=a{\left(|\cos(\frac{m}{4}\theta ){|}^{n_{2}}+|\frac{1}{k}\sin(\frac{m}{4}\theta ){|}^{n_{3}}\right)}^{-\frac{1}{n_{1}}},$$

where *r* and *θ* represent the polar radius and polar angle, respectively; *a*, *k*, *n*_1_, *n*_2_ and *n*_3_ are parameters to be fitted; and *m* is a positive integer that determines the number of angles of the Gielis curve within the interval [0, 2π) (Wang et al., 2022a). In recent years, various studies have demonstrated the validity of the Gielis equation for describing actual biological geometries, e.g., leaf shapes (Lin et al., 2016; Shi et al., 2018, 2019; Yao et al., 2022), planar projections of seed and fruit (Tian et al., 2020; Yao et al., 2024), geometries of the outer rims of corolla tubes (Wang et al., 2022b), and shapes of some sea stars (Shi et al., 2020), as well as egg shapes of birds (Shi et al., 2022a). Particularly, bamboo leaves—with their easy accessibility and clear morphological boundaries—have become a preferred material for validating the Gielis equation in studies of natural geometries (Shi et al., 2015a; Yao et al., 2022). Shi et al. (2015a) employed a simplified Gielis equation with two parameters to describe the shape of bamboo leaves, in which one parameter represented the overall ratio of leaf width to leaf length. Yao et al. (2022) compared a three-parameter Gielis equation with a two-parameter version using leaf boundary coordinate data from six bamboo species within the same genus, all characterized by distinct long lanceolate leaves. Their study aimed to determine whether the three-parameter Gielis equation could enhance the model’s fitting accuracy for bamboo leaf shapes.

However, previous researches on model evaluation have primarily focused on assessing goodness of fit (e.g., the coefficient of determination) or examining the trade-off between goodness of fit and model complexity (e.g., the Akaike information criterion) (Shi et al., 2015a; Lin et al., 2016; Yao et al., 2022). Despite its potential to offer valuable insights into plant leaf formation mechanisms, the nonlinearity of the Gielis equation has remained largely unexplored in terms of quantification and comparison using relative curvature measures of nonlinearity. In fact, relative curvature measures of nonlinearity offer a more comprehensive evaluation of the nonlinear behavior of models such as the Gielis equation, providing insights beyond traditional criteria like the coefficient of determination or the Akaike information criterion. While conventional metrics primarily assess how well a model fits the data, they fail to capture the intrinsic nonlinearity of the model and its potential impact across varying datasets or conditions. However, relative curvature measures quantify how closely a nonlinear regression model approximates linear behavior (Bates and Watts, 1980), which is particularly relevant for Gielis curves derived from the Gielis equation, where nonlinearity is an inherent characteristic. By incorporating these nonlinear measures, studies can ensure that selected models not only achieve better fit to the data but also offer a deeper understanding of their structural properties. This makes them particularly valuable for comparing nonlinear models, especially in ecological and biological research, where nonlinearity is a fundamental feature.

To systematically investigate the intrinsic nonlinearity of the Gielis equation, we employed three simplified versions (i.e., the four-parameter, three-parameter, and two-parameter Gielis equations) to model leaf contours of two bamboo species within the same genus, both exhibiting characteristic lanceolate morphology. Each species had a dataset of more than 250 leaves, ensuring robust statistical analysis. Model performance was evaluated using root-mean-square error, the Akaike information criterion, and relative curvature measures of nonlinearity to identify which of the three nonlinear models best captured the geometric properties of bamboo leaves. This study aims to validate the effectiveness of relative curvature measures of nonlinearity in nonlinear regression analysis and to introduce a novel approach for assessing the Gielis equation’s suitability in describing the geometries of natural plants. Our work establishes curvature analysis as a vital complement to conventional model selection criteria in plant morphometrics.

## 2 Materials and methods

### 2.1 Leaf collection

**Figure 1:**
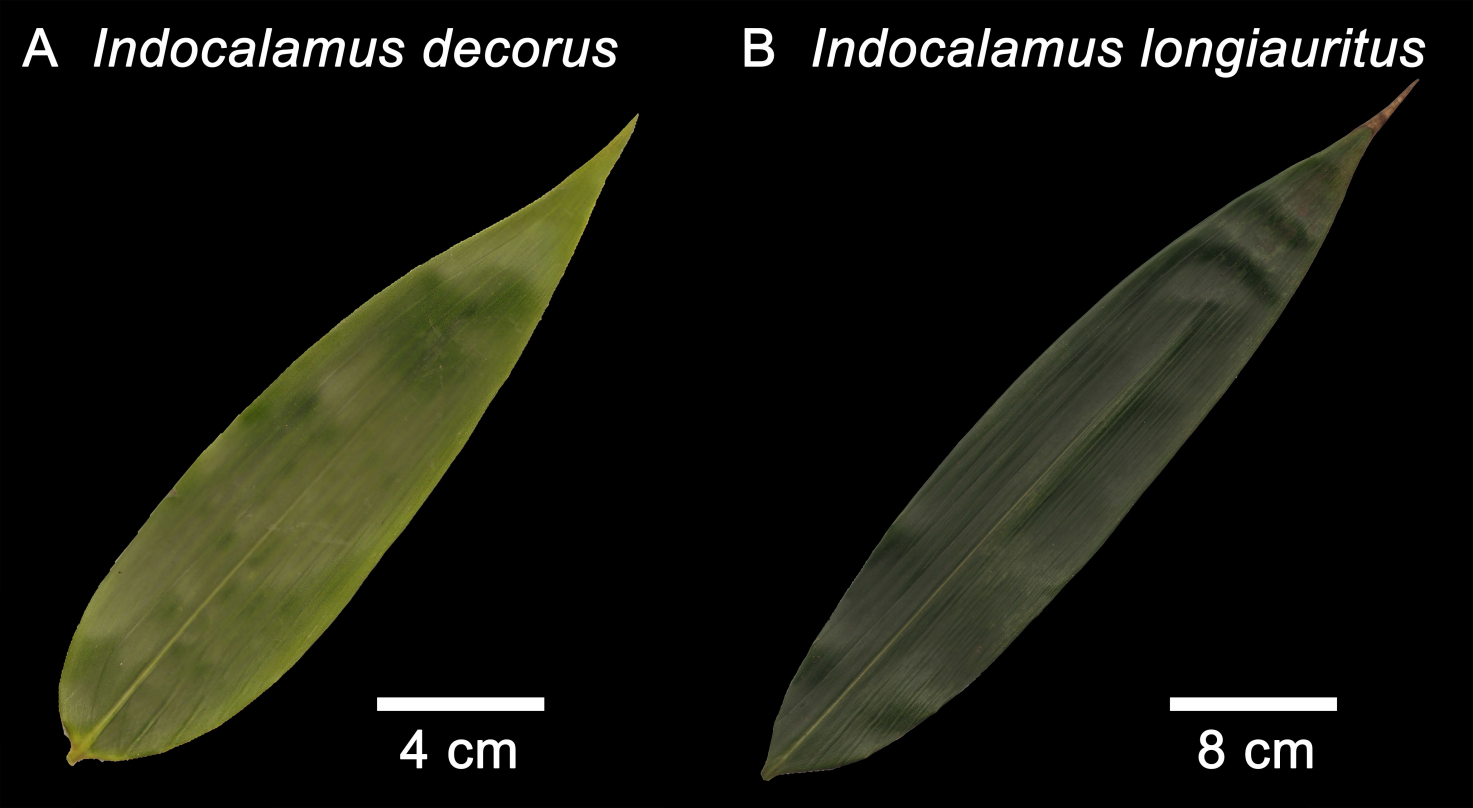
Outlines of leaf samples of **(A)** *Indocalamus decorus* and **(B)** *Indocalamus longiauritus*.

During November 2024, we randomly collected 254 mature leaves from 122 healthy culms of *Indocalamus decorus* in Yuping Town, Hongya County, Sichuan Province, China (103°27’53’’E, 29°55’33’’N). Similarly, in November 2024, an additional 251 leaves were sampled from 120 culms of *Indocalamus longiauritus* at the Bamboo Resource Base of Leshan Normal University, Leshan City, Sichuan Province, China (103°44’57’’E, 29°33’54’’N). To preserve fresh weight and minimize morphological distortion, all leaves of each species were promptly wrapped in wet paper and transported to the laboratory within 2 hours post-collection. Although variations in sampling vertical positions, azimuth angles, leaf age, and culm age were present, their potential effects were statistically negligible given our large sample size (more than 250 per species). For each species, leaves were randomly collected from at least 120 healthy culms, encompassing a wide range of canopy positions and orientations. Sampling occurred near the end of the second growth season, ensuring that all shoots were mature and the collected leaves had fully expanded. This sampling strategy—randomized and distributed across individuals and canopy layers—was intended to minimize potential biases associated with developmental stage or microenvironmental variation (Shi et al., 2015a; Lin et al., 2016; Yao et al., 2022). **Figure 1** provides representative leaf profiles of *I. decorus* and *I. longiauritus*.

### 2.2 Data acquisition

Fresh leaves were scanned using a photo scanner (M208, BenQ, Shanghai, China) at a resolution of 600 dpi and saved as PDF files. To minimize scanning-related distortions, the surface moisture of each leaf was gently wiped dry prior to scanning to ensure clean image boundaries. Additionally, leaves were carefully flattened during placement on the scanner to avoid folding or curvature, thereby preserving their natural two-dimensional geometry. Subsequently, Adobe Photoshop CS2 (version 9.0; Adobe, San Jose, CA, USA) was employed to convert the PDF images into black and white images saved as BMP files for each individual leaf. Planar leaf boundaries were extracted using a custom MATLAB script (version ≥ 2009a; MathWorks, Natick, MA, USA) following the methodology described in Shi et al. (2018) and Su et al. (2019). These procedures have been demonstrated to yield reliable and reproducible results in extracting leaf boundary data for geometric analysis (Yu et al., 2020; Guo et al., 2021; Wang et al., 2024a). Each leaf boundary was represented by approximately 2000 equidistant points, extracted using the “adjdata” function from the “biogeom” package (version 1.4.3; Shi et al., 2022b). The raw data for leaf boundary coordinates are accessible in online **Supplementary Tables S1** and **S2**.

### 2.3 Models

To balance model flexibility and computational efficiency for fitting bamboo leaf boundaries, we employed a simplified version of the Equation 1 by setting *k* = *m* = 1, following established methodologies (Shi et al., 2015a; Wang et al., 2022a). This four-parameter formulation (denoted as SGE-1) is expressed as


(2) $$r(\theta )=a{\left(|\cos(\frac{1}{4}\theta ){|}^{n_{2}}+|\sin(\frac{1}{4}\theta ){|}^{n_{3}}\right)}^{-\frac{1}{n_{1}}},$$

where parameter *n*_1_, *n*_2_, and *n*_3_ can assume distinct values, enabling generation of both symmetrical and asymmetrical leaf geometries. The parameter *a* primarily controls the overall scaling of the leaf shape and correlates strongly with leaf area (Gielis, 2003). Parameter *n*_1_ is the principal shape determinant and has been shown to be positively associated with the width-to-length ratio of the leaf (Yao et al., 2022). Parameters *n*_2_ and *n*_3_ collectively define the symmetry characteristics of the leaf contour; when *n*_2_ and *n*_3_ are equal, the resulting shape exhibits perfect bilateral symmetry (Wang et al., 2022a). While SGE-1 offers greater shape diversity, its computational demands increase with parameter dimensionality.

For cases requiring more symmetrical shape representation, we adopted the three-parameter reduction (denoted as SGE-2) proposed by Yao et al. (2022), where *n*_3_ = *n*_2_ in Equation 2:


(3) $$r(\theta )=a{\left(|\cos(\frac{1}{4}\theta ){|}^{n_{2}}+|\sin(\frac{1}{4}\theta ){|}^{n_{2}}\right)}^{-\frac{1}{n_{1}}}.$$

Further simplification was achieved through the two-parameter version developed by Shi et al. (2015a), setting *n*_2_ = 1 in Equation 3:


(4) $$r(\theta )=a{\left(|\cos(\frac{1}{4}\theta )|+|\sin(\frac{1}{4}\theta )|\right)}^{-\frac{1}{n_{1}}}.$$

Equation 4 was denoted as SGE-3. Notably, the SGE-3 has demonstrated excellent fitting performance for empirical bamboo leaf boundary coordinate data despite its reduced number of parameters (Shi et al., 2015a; Lin et al., 2016; Yao et al., 2022), making it particularly suitable for large-scale analyses.

### 2.4 Data fitting and model evaluation

The three nonlinear models (SGE-1, SGE-2, and SGE-3; Section 2.3) were employed to fit empirical leaf boundary coordinate data utilizing the Nelder-Mead optimization algorithm (Nelder and Mead, 1965) within a general-purpose framework. The Nelder-Mead algorithm was selected because of its simplicity, robustness in low-dimensional parameter spaces, and ability to handle non-differentiable or noisy objective functions. Given the limited number of parameters (2–4 in the tested models), the algorithm has been shown to perform effectively in similar morphological modeling tasks. Previous studies (e.g., Lin et al., 2016; Shi et al., 2022a) have successfully employed the Nelder-Mead method to estimate parameters of simplified Gielis equation with high fitting accuracy in plant geometry studies. To reduce the risk of convergence to local optima, we performed multiple optimization runs with different initial parameter values for each individual leaf profile. Illustrative R scripts for implementing the data fitting procedure using the simplified Gielis equation is available in **Supplementary Material S4** of Shi et al. (2018).

Parameters of each simplified Gielis equation were estimated by minimizing the residual sum of squares (RSS) between the observed and predicted radii from the polar point to the leaf boundary:


(5) $$\text{RSS}=\sum_{i=1}^{N}{\left(r_{i}-{\hat{r}}_{i}\right)}^{2},$$

where *r_i_* in Equation 5 represents the observed distance from the polar point to the *i*-th point on the scanned perimeter of leaf shape; ${\hat{r}}_{i}$ represents the predicted distance from the polar point to the *i*-th point on the predicted perimeter of leaf shape based on the each simplified Gielis equation; and *N* is the number of data points on the scanned perimeter of leaf shape. The root-mean-square error (RMSE) was calculated to evaluate the goodness of fit of the nonlinear regression:


(6) $$\text{RMSE}=\sqrt{\text{RSS}/\left(N-P\right)},$$

where *P* in Equation 6 is the number of parameters for each simplified Gielis equation. The smaller RMSE value, the better the model fits. Additionally, we computed the Akaike information criterion (AIC) to balance the trade-off between goodness of fit and model structural complexity (Burnham and Anderson, 2004). The model with the lowest AIC value is considered best. Wilcoxon signed rank test (Wilcoxon, 1945) with a 0.05 significance level was employed to determine whether there were significant differences among RMSE or AIC values derived from different models.

When applying least squares protocols to fit a mathematical model, it is essential to consider the stochastic assumption about the random error term (Bates and Watts, 1980). This involves specifying the nature of the error term, which in this case captures the differences between the observed and predicted polar radii as they vary with changes in the polar angle. Under the classical assumption that these errors are independently and identically distributed following a normal distribution, the least squares estimators in linear regression are known to be unbiased, jointly normally distributed, and exhibit minimum variance among estimators within the class of regular estimators (Ratkowsky and Reddy, 2017). However, these guarantees often fail in nonlinear contexts. In such cases, particularly when the sample size is limited, least squares estimators may be biased and inefficient. As a result, assessing the validity of the underlying assumptions for the three nonlinear models (i.e., SGE-1, SGE-2, and SGE-3) is a critical component of the analysis.

The core principle behind most algorithms for estimating parameters using least squares in nonlinear models and many associated inference techniques is the use of a first-order Taylor series expansion to locally approximate the nonlinear function with a linear one (Bates and Watts, 1980, 1988). The linear approximation corresponds to two distinct assumptions: the planar assumption and the uniform coordinate assumption (Bates and Watts, 1980). A variety of measures of nonlinearity have been developed to evaluate how well a linear approximation captures the behavior of a nonlinear model, or to reveal its limitations when it does not, i.e., confidence regions (Beale, 1960), bias (Box, 1971), skewness (Hougaard, 1985), and kurtosis (Haines et al., 2004). The root-mean-square relative curvatures ( ${\text{γ}}_{\text{RMS}}$ ) (Bates and Watts, 1980; He et al., 2024), including the root-mean-square relative intrinsic curvature ( ${\text{γ}}_{\text{RMS}}^{N}$ ) and the root-mean-square relative parameter-effects curvature ( ${\text{γ}}_{\text{RMS}}^{T}$ ), offers comprehensive evaluations to determine whether a nonlinear regression model aligns “close-to-linear” or “far-from-linear”. A “close-to-linear” model means that a nonlinear model has least squares estimators closely approached the mentioned asymptotic properties (i.e., unbiased, jointly normally distributed, and exhibit minimum variance) (Ratkowsky, 1983, 1990). In contrast, “far-from-linear” nonlinear models lacked these desirable asymptotic properties. The two root-mean-square relative curvatures ${\text{γ}}_{\text{RMS}}^{N}$ and ${\text{γ}}_{\text{RMS}}^{T}$ were evaluated by the critical curvature (*K_c_*), defined as $1/\sqrt{F(P,N-P;\text{α})}$ , where *F* represents the *F*-distribution, *P* is the number of the model parameters, *N* is the number of data points, and α is the confidence level equal to 0.05 (Bates and Watts, 1980; He et al., 2024). Here, a value of ${\text{γ}}_{\text{RMS}}^{N}$ is smaller than *K_c_* suggests that the planar assumption is acceptable. Meanwhile, if ${\text{γ}}_{\text{RMS}}^{T}$ value is smaller than *K_c_*, then the uniform coordinate assumption holds true. Indeed, it is common for most nonlinear regression models to exhibit relatively low ${\text{γ}}_{\text{RMS}}^{N}$ values, which often fall below the critical threshold, as intrinsic curvature primarily reflects the overall geometric nonlinearity of the model structure. In contrast, ${\text{γ}}_{\text{RMS}}^{T}$ is more sensitive to the behavior of individual model parameters and typically yields higher values, making it more challenging to meet the close-to-linear criteria (Ratkowsky and Reddy, 2017; He et al., 2024).

While ${\text{γ}}_{\text{RMS}}^{N}$ and ${\text{γ}}_{\text{RMS}}^{T}$ provide valuable global assessments of model nonlinearity, they offer limited insight into individual model parameter performance on the linear approximation. To address this limitation, we complemented our analysis by employing the percentage bias (*P_b_*) of each parameter, as suggested by Box (1971) and Ratkowsky (1983), to evaluate the nonlinear behavior for a particular parameter within a nonlinear model. As a general guideline, when the absolute value of *P_b_* falls below 1%, the nonlinear model exhibits “close-to-linear” behavior. This suggests that the parameter estimators possess several desirable asymptotic characteristics, including proximity to unbiasedness, normal distribution, and minimization of variance (Ratkowsky, 1990).

The function “fitGE” from the “biogeom” package (version 1.4.3; Shi et al., 2022b) were used to estimate the model parameters within the three simplified Gielis equation (i.e., SGE-1, SGE-2, and SGE-3). The functions “curvIPEC” and “biasIPEC” from the “IPEC” package (version 1.1.0; Shi et al., 2024) were used to calculate the curvature measures of nonlinearity described above, including ${\text{γ}}_{\text{RMS}}^{N}$ , ${\text{γ}}_{\text{RMS}}^{T}$ , *K_c_*, and *P_b_*. All calculations and figures were accomplished based on R (version 4.2.1; R Core Team, 2022).

## 3 Results

**Figure 2:**
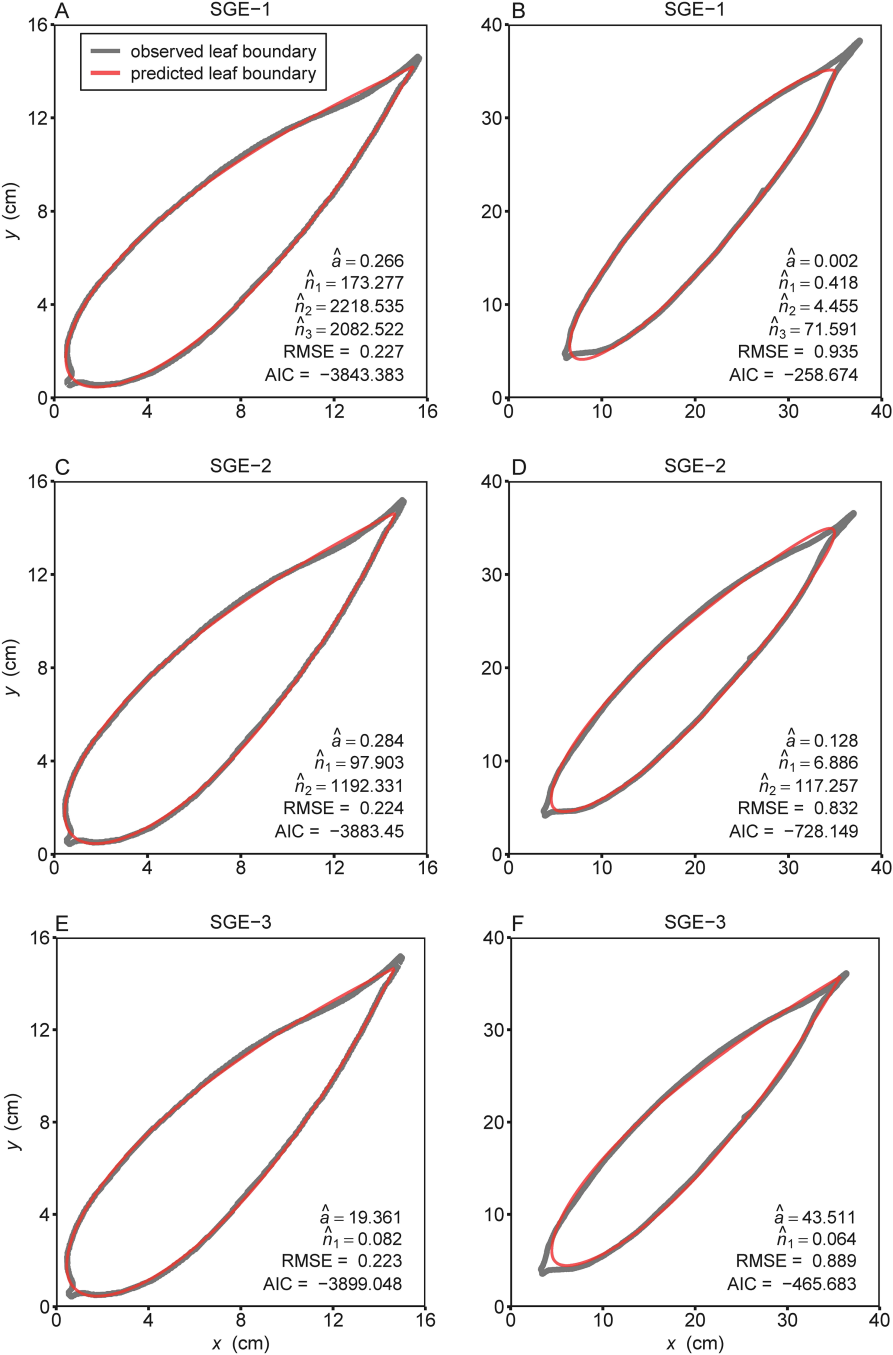
The observed (gray curves) and predicted (red curves) boundary geometries of the representative leaves of **(A, C, E)** *Indocalamus decorus* and **(B, D, F)** *Indocalamus longiauritus* (see **Figure 1**) simulated using the three simplified Gielis equations (SGE-1, SGE-2, and SGE-3). Letters *a*, *n*_1_, *n*_2_, and *n*_3_ with hats represent the estimated values of parameters of the corresponding simplified Gielis equation in each panel; RMSE represents the root-mean-square error; AIC represents the Akaike information criterion.

**Figure 3:**
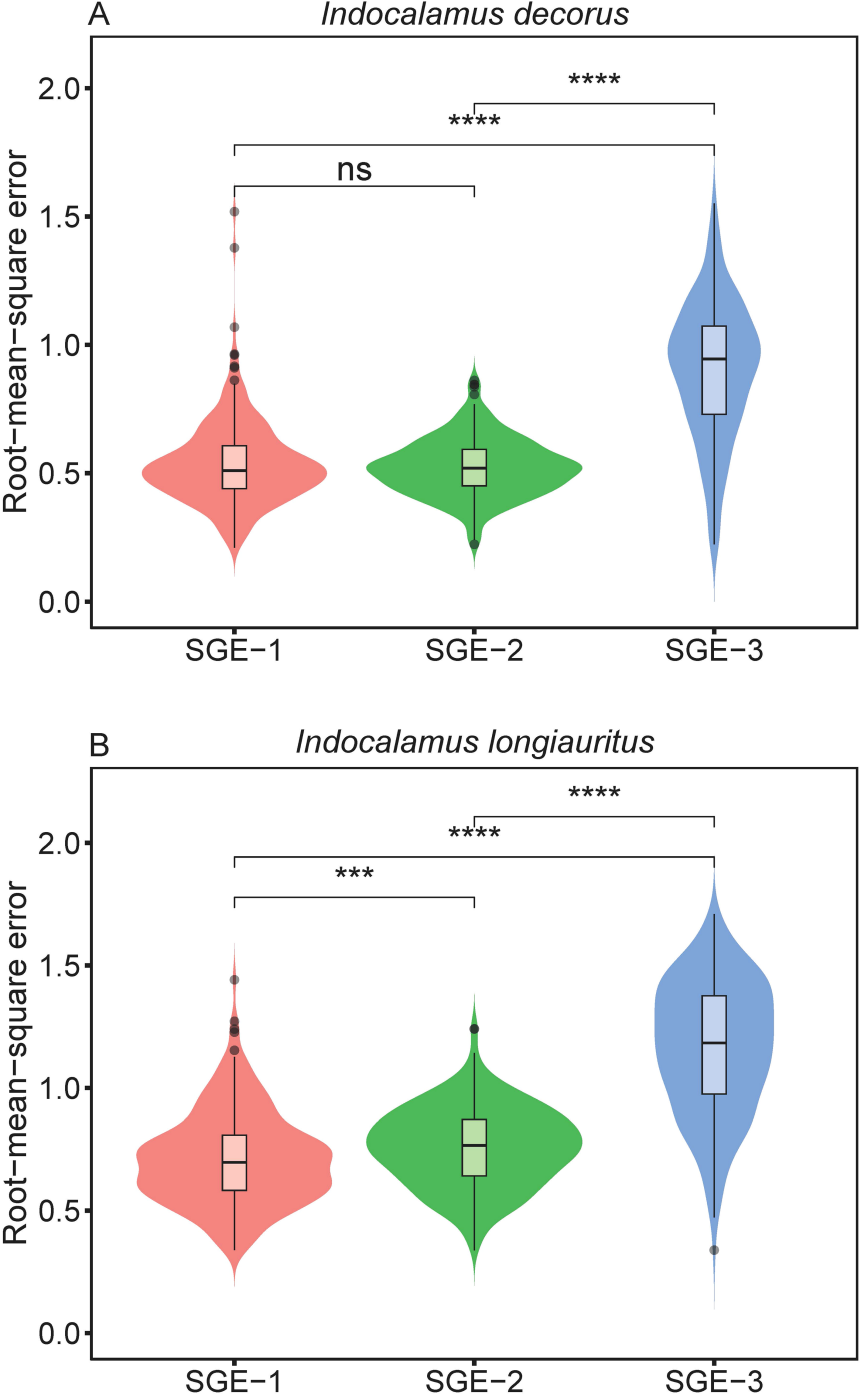
Violin plots display the distribution of the root-mean-square error values derived from the three simplified Gielis equations (SGE-1, SGE-2, and SGE-3). Horizontal bar within each box denotes medians; bottoms and tops of boxes represent 25th and 75th percentiles, and lines extend to the 1.5-fold interquartile range. Statistical significance of the two-sided Wilcoxon test at the 0.05 significance level is marked: ns for not significant, *** for p < 0.001, and **** for p < 0.0001.

The three models (i.e., SGE-1, SGE-2, and SGE-3) generally provided effective representations to the boundary of bamboo leaves for both species (**Supplementary Tables S3–S5** in the **Supplementary Materials**). **Figure 2** illustrates the fitting results of the leaf profile using the three models for two leaf examples as intuitively shown in **Figure 1**. For species of *I. decorus*, the results of Wilcoxon signed rank test for every two models, conducted at a significance level of 0.05, revealed that SGE-3 exhibited significantly highest RMSE values than other two models. While there was no significant difference in RMSE values derived from SGE-1 and SGE-2 (**Figure 3**; **Supplementary Table S6**). For species of *I. longiauritus*, the results of Wilcoxon signed rank test for every two models indicated that SGE-3 exhibited significantly highest RMSE values and SGE-1 exhibited significantly lowest RMSE values (**Figure 3**; **Supplementary Table S6**). These findings suggest that SGE-1 demonstrated the best goodness of fit for both species.

**Figure 4:**
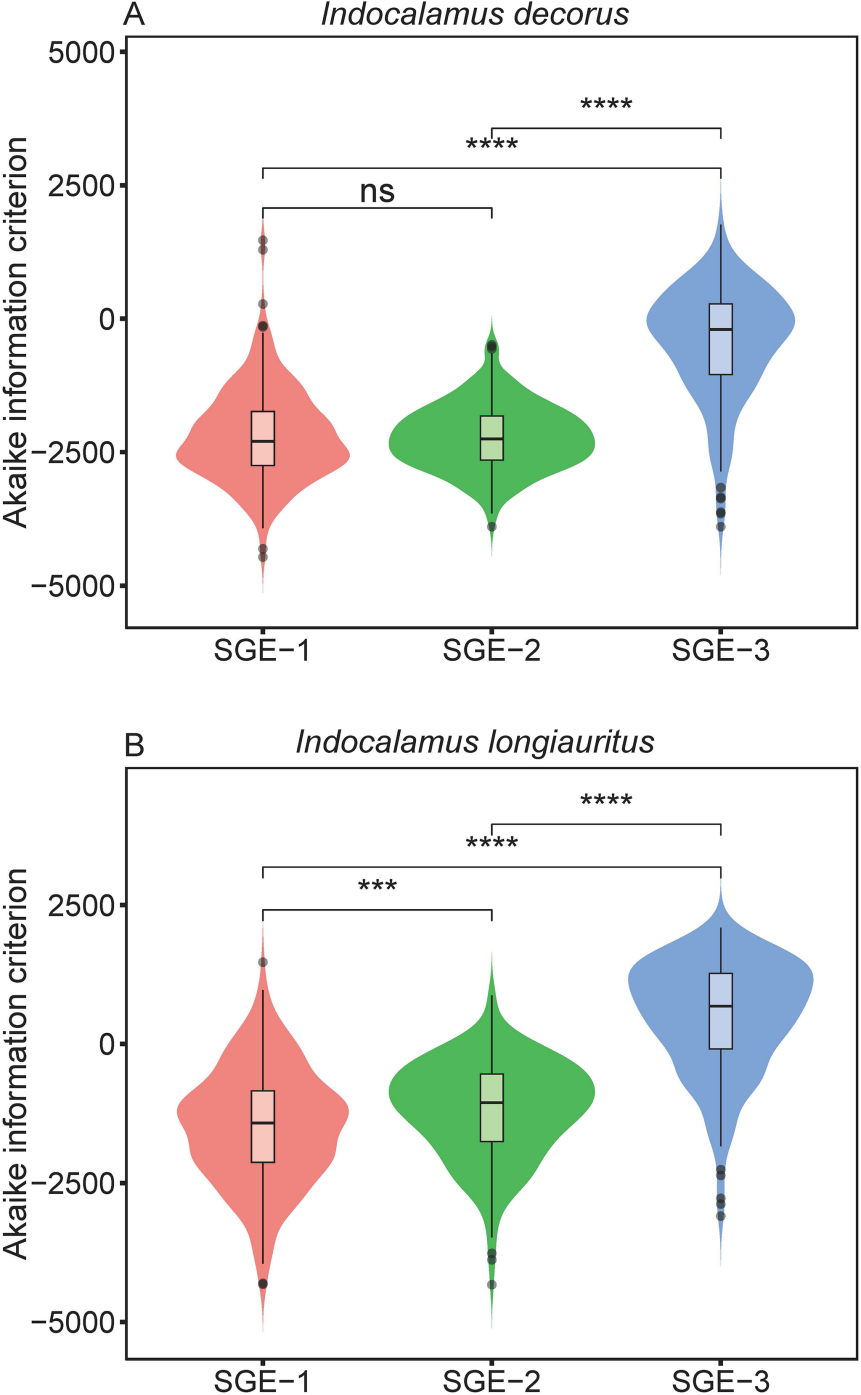
Violin plots display the distribution of the Akaike information criterion values derived from the three simplified Gielis equations (SGE-1, SGE-2, and SGE-3). Horizontal bar within each box denotes medians; bottoms and tops of boxes represent 25th and 75th percentiles, and lines extend to the 1.5-fold interquartile range. Statistical significance of the two-sided Wilcoxon test at the 0.05 significance level is marked: ns for not significant, *** for p < 0.001, and **** for p < 0.0001.

Furthermore, the Wilcoxon signed rank tests revealed that SGE-3 had the highest AIC values among the three models for the empirical data of both species at a significance level of 0.05 (**Figure 4**; **Supplementary Table S7**). And for species of *I. decorus*, there was no significant difference in AIC values derived from SGE-1 and SGE-2; for species of *I. longiauritus*, SGE-1 exhibited significantly lower AIC values than SGE-2 (**Figure 4**; **Supplementary Table S7**). These results indicate that SGE-1 outperformed the other models in nonlinear regression by achieving a favorable trade-off between model structural complexity and goodness of fit.

**Figure 5:**
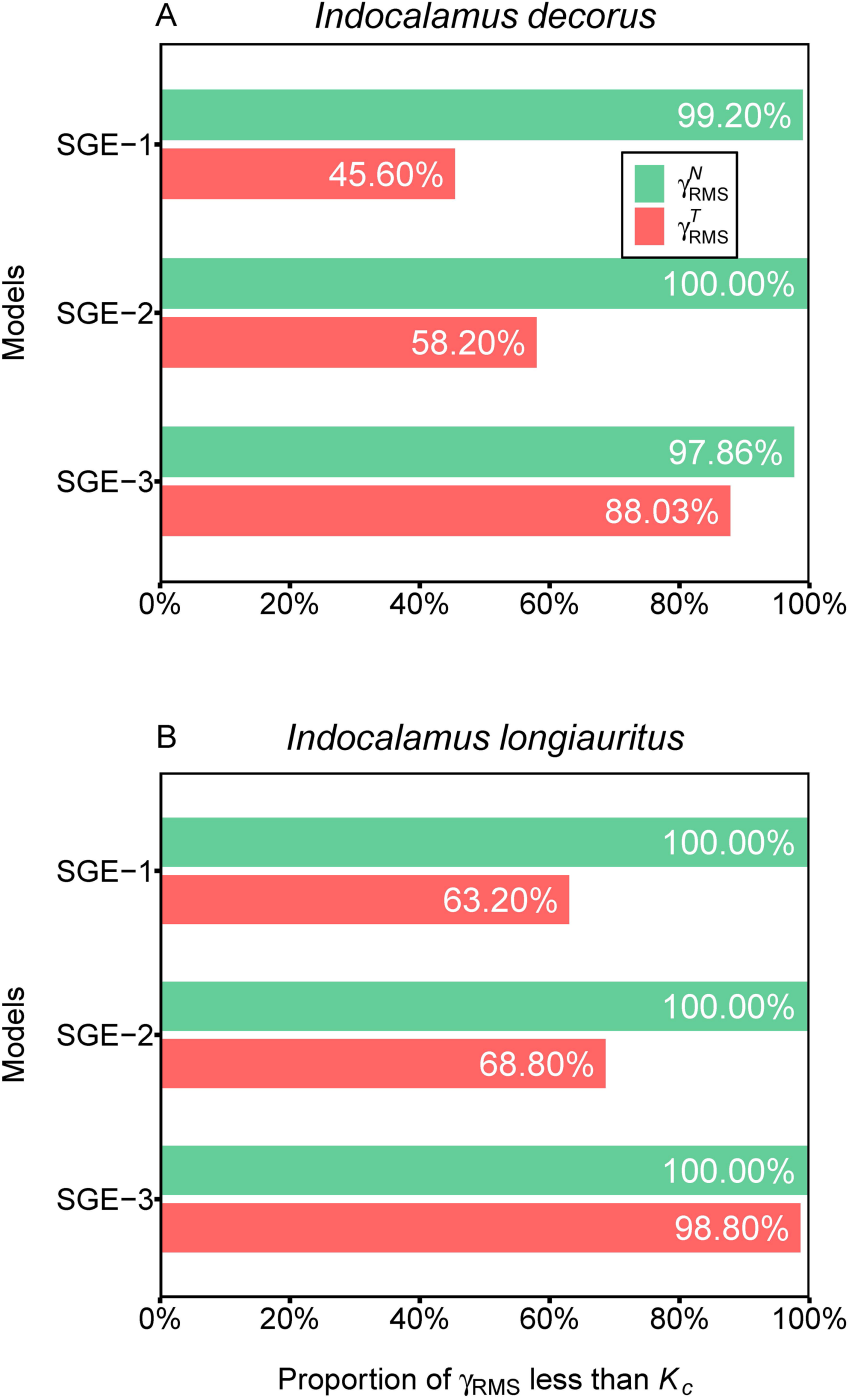
Assessment of nonlinear behavior of the three simplified Gielis equations (SGE-1, SGE-2, and SGE-3) at the global level for two datasets. ${\text{γ}}_{\text{RMS}}^{N}$ represents root-mean-square relative intrinsic curvature, ${\text{γ}}_{\text{RMS}}^{T}$ represents root-mean-square relative parameter-effects curvature, and *K_c_* represents critical curvature. For example, 99.20% in **(A)** represents for SGE-1, there are 99.20% of ${\text{γ}}_{\text{RMS}}^{N}$ values which are smaller than the corresponding *K_c_* for *Indocalamus decorus*; 68.80% in **(B)** represents for SGE-2, there are 68.80% of ${\text{γ}}_{\text{RMS}}^{T}$ values that are smaller than the corresponding *K_c_* for *Indocalamus longiauritus*.

The overall nonlinearity of the nonlinear regression models was evaluated by the root-mean-square relative curvatures, i.e., ${\text{γ}}_{\text{RMS}}^{N}$ , ${\text{γ}}_{\text{RMS}}^{T}$ , and *K_c_*. For *I. decorus* leaf data across the three models, 99.20% (SGE-1), 100% (SGE-2), and 97.86% (SGE-3) of the 254 leaves had ${\text{γ}}_{\text{RMS}}^{N}$ values smaller than the corresponding *K_c_*. Additionally, the proportions of ${\text{γ}}_{\text{RMS}}^{T}$ values less than the corresponding *K_c_* were 45.60% (SGE-1), 58.20% (SGE-2), and 88.03% (SGE-3) (**Figure 5A**; **Supplementary Tables S3-S5**). For *I. longiauritus* leaf data, all of the ${\text{γ}}_{\text{RMS}}^{N}$ values were less than the corresponding *K_c_* across the three models. The proportions of ${\text{γ}}_{\text{RMS}}^{T}$ values smaller than the corresponding *K_c_* were 63.20% (SGE-1), 68.80% (SGE-2), and 98.80% (SGE-3) (**Figure 5B**; **Supplementary Tables S3–S5**).

These results indicated that SGE-3 exhibited the best linear approximation among the three models, while the SGE-1 showed the worst performance in linear approximation. Notably, all three models demonstrated exceptional adherence to the planar assumption, with over 97% of ${\text{γ}}_{\text{RMS}}^{N}$ values being smaller than the corresponding *K_c_* in both species. Regarding the uniform coordinate assumption, the SGE-3 emerged as the most satisfactory among all models, as evidenced by over 88% of ${\text{γ}}_{\text{RMS}}^{T}$ values being less than the corresponding *K_c_* in both species.

**Figure 6:**
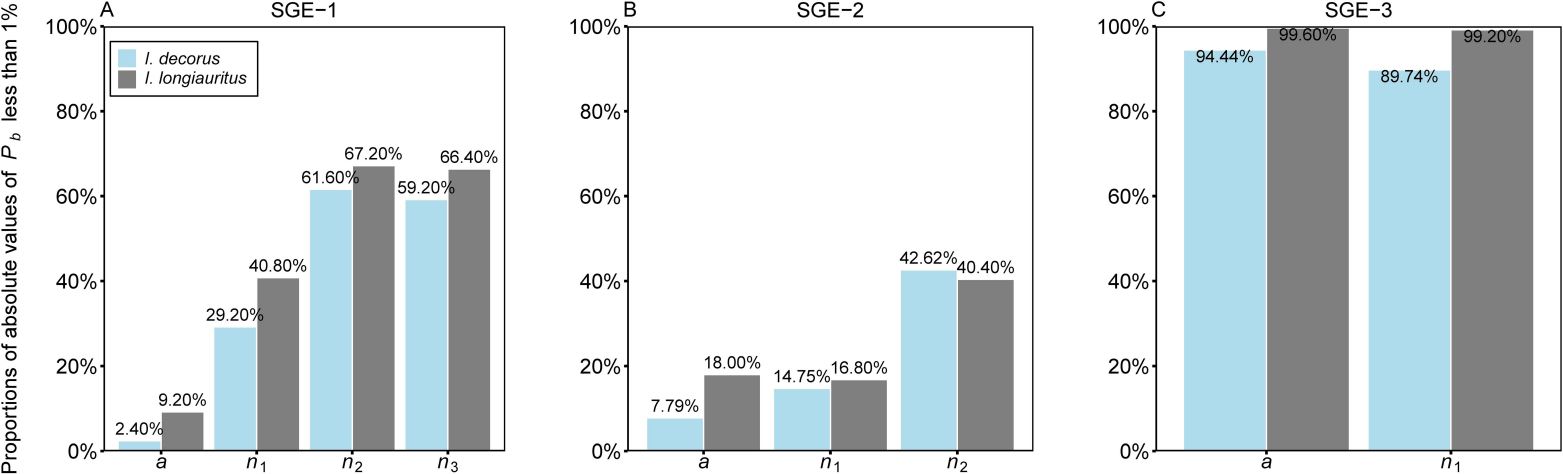
Bar chart of the proportions of the absolute values of percentage bias (*P_b_*) of each parameter in the three simplified Gielis equations (**(A)** SGE-1, **(B)** SGE-2, and **(C)** SGE-3) less than 1% for two datasets. For example, 2.40% in **(A)** represents there are 2.40% of the absolute values of *P_b_* of parameter *a* of SGE-1, which are smaller than 1% for the data of *Indocalamus decorus*; 9.20% in **(A)** represents there are 9.20% of the absolute values of *P_b_* of parameter *a* of SGE-1, which are smaller than 1% for the data of *Indocalamus longiauritus*.

In terms of individual parameter-level nonlinear behavior, examining the percentage bias (*P_b_*) of parameters reveals insightful findings. For SGE-1, the absolute values of *P_b_* for *a*, *n*_1_, *n*_2_, and *n*_3_ were smaller than 1% in 2.40%, 29.20%, 61.60%, and 59.20% of cases, respectively, in the data of *I. decorus* leaves. Correspondingly, these proportions were 9.20%, 40.80%, 67.20%, and 66.40% for the data of *I. longiauritus* leaves (**Figure 6A**; **Supplementary Table S3**). Moving to SGE-2, 7.79%, 14.75%, and 42.62% of the absolute values of *P_b_* for *a*, *n*_1_, and *n*_2_ were below 1% for *I. decorus*, and these figures were 18.00%, 16.80%, and 40.40% for *I. longiauritus* (**Figure 6B**; **Supplementary Table S4**). Regarding SGE-3, 94.44%, and 89.74% of the absolute values of *P_b_* for *a*, and *n*_1_, respectively, were below 1% in *I. decorus* leaves data, and 99.60%, and 99.20% in *I. longiauritus* leaves data (**Figure 6C**; **Supplementary Table S5**).

These results underscored that SGE-3 exhibited the best close-to-linear behavior among the three models, with over 89% of the absolute values of *P_b_* for each parameter being smaller than 1% in both species. SGE-1 demonstrates relatively good close-to-linear behavior, except for parameter *a*, where <10% of the absolute values of *P_b_* are less than 1%. However, SGE-2 performs poorly, with three parameters (*a*, *n*_1_, and *n*_2_) having less than 43% of their absolute values of *P_b_* below 1% for both datasets.

## 4 Discussion

The Gielis equation, originating from the superellipse formulation (Lamé, 1818), has demonstrated remarkable versatility in modeling diverse biological morphologies. Previous applications include: (1) simplified versions for characterizing tree ring cross-sections in conifers (Shi et al., 2015a, 2015b) and leaf boundaries across 46 bamboo species (Lin et al., 2016); (2) modified twin versions showing superior performance in describing the morphologies of some sea stars (Shi et al., 2020); and (3) successful applications in quantifying seed projections in *Ginkgo biloba* cultivars (Tian et al., 2020) and fruit geometries in *Koelreuteria paniculata* (Li et al., 2022). Notably, Wang et al. (2022b) extended its utility to corolla tube geometries in *Vinca major*. These collective findings underscore the equation’s adaptability, though further validation across morphologically diverse specimens within single taxa remains valuable.

Traditional model evaluation metrics (e.g., the goodness of fit and the Akaike information criterion) face limitations when assessing complex nonlinear models with multiple parameters (Ratkowsky and Reddy, 2017). Beyond mere fitting accuracy, an ideal nonlinear model should ensure parameter estimators exhibit close-to-linear behavior including unbiasedness, normality, and minimum variance (Ratkowsky, 1990; Ratkowsky and Reddy, 2017). Our comprehensive evaluation framework addresses these requirements through simultaneous consideration of both fitting performance and intrinsic nonlinearity characteristics.

Our analysis reveals distinct trade-offs among the three nonlinear models scrutinized in this study. We found that SGE-1 demonstrated superior fitting performance, as illustrated in **Figure 3**. Meanwhile, SGE-1 exhibited a robust performance in adhering to the planar assumption, with almost all of ${\text{γ}}_{\text{RMS}}^{N}$ values being smaller than the corresponding *K_c_* in both species (**Figure 5**). However, SGE-1 showed limitations in confirming the uniform coordinate assumption, as over 36% of ${\text{γ}}_{\text{RMS}}^{T}$ values were greater than the corresponding *K_c_* in both species (**Figure 5**). Particularly, only two out of four parameters within SGE-1, namely *n*_2_ and *n*_3_, demonstrated close-to-linear behavior, as assessed by metrics such as lower percentage bias (**Figure 6**). For the other end of the goodness of fit scale, SGE-3 was found to be poor in fitting the observed data (**Figure 3**). Despite its shortcomings in goodness of fit, SGE-3 demonstrated commendable performance in terms of being close-to-linear. To be specific, SGE-3 had the best linear approximation (**Figure 5**). And at the individual parameter level, all parameters of SGE-3, *a* and *n*_1_, were close-to-linear as judged by the lower percentage bias (**Figure 6**).

After assessing the nonlinear regression models using various methods, it can be concluded that SGE-3 exhibited the poorest goodness of fit and the highest AIC values, whereas SGE-1 showcased the best goodness of fit and the lowest AIC values. However, despite SGE-3’s suboptimal goodness of fit, it demonstrated the best close-to-linear behavior among the three models, both as an overall measure and at the individual parameter level of nonlinear behavior. On the other hand, SGE-1 faced challenges related to the uniform coordinate assumption, and displayed drawbacks in the behavior of four of its parameters, with two of them, i.e., *a*, and *n*_1_, were not close-to-linear. Therefore, among the three models examined, SGE-1 emerges as the clear choice when considering mainly the goodness of fit and AIC. While if one focuses on the nonlinear behavior, SGE-3 might be considered the optimal selection. It is essential to note that future studies on different species may lead to different conclusions. The choice between models should therefore be guided by study objectives—prioritizing either morphological characterization or statistical inference.

Generally, through an appropriate nonlinear reparameterization can effectively reduce parameter-effects curvature in nonlinear models (Bates and Watts, 1980; Ratkowsky and Reddy, 2017). It has been confirmed that performing specific parameter transformations like exponential modification in nonlinear regression models can simultaneously improve the parameter-effects curvature and close-to-linear behavior of the model parameters (He et al., 2024; Wang et al., 2024b). However, such approaches risk increasing model complexity, particularly problematic for already intricate formulations like the Gielis equation. Based on this trade-off analysis, we recommend retaining the current parameterization while identifying this as a promising direction for future methodological research.

## 5 Conclusions

In summary, we evaluated three simplified Gielis equations for modeling leaf contours of two bamboo species. The results derived from the two datasets demonstrated a clear trade-off between model performance metrics: while SGE-3 had the best close-to-linear behavior among the three models, it exhibited the poorest goodness of fit and the highest AIC value. In contrast, although SGE-1 achieved the best fit quality and the lowest AIC value, it provided unacceptable close-to-linear least squares estimates of parameters. Consequently, the choice of model applied for capturing the geometric properties of bamboo leaves depends on the criteria used to evaluate it, e.g., goodness of fit, model structural complexity, and the close-to-linear behavior of parameters. When models exhibit comparable fit, assessing parameter nonlinearity becomes critical for optimal selection. The present work provided insights into the criteria of model selection for nonlinear regression for future researches on describing the leaf shape of bamboo and other plant species with similar lanceolate leaves. One limitation of the current work is that it does not address the ecological or functional significance of the lanceolate leaf shape in bamboo, such as its potential role in drought resistance or other environmental adaptations. Additionally, the relationship between the parameters of the simplified Gielis equations and specific ecological traits remains unclear. Future research should aim to bridge this gap by linking shape descriptors derived from geometric models with physiological and ecological functions. Such studies would deepen the understanding of how leaf morphology contributes to adaptive strategies in varying environments and enhance the applicability of geometric modeling in functional plant ecology.

## Data Availability

The original contributions presented in the study are included in the article/**Supplementary Material**. Further inquiries can be directed to the corresponding authors.
